# Supplementary material for: Venomix: a simple bioinformatic pipeline for identifying and characterizing toxin gene candidates from transcriptomic data
Source: PeerJ. 2018 Jul 31;6:e5361. doi: 10.7717/peerj.5361 (PMC6074769; doi:10.7717/peerj.5361)
Supplement: Supplemental Information 3 [file peerj-06-5361-s003.gz › FinalOutput_E-6/Kunitz-type_serine_protease_inhibitor_bicolin_1/finaltree.pdf]

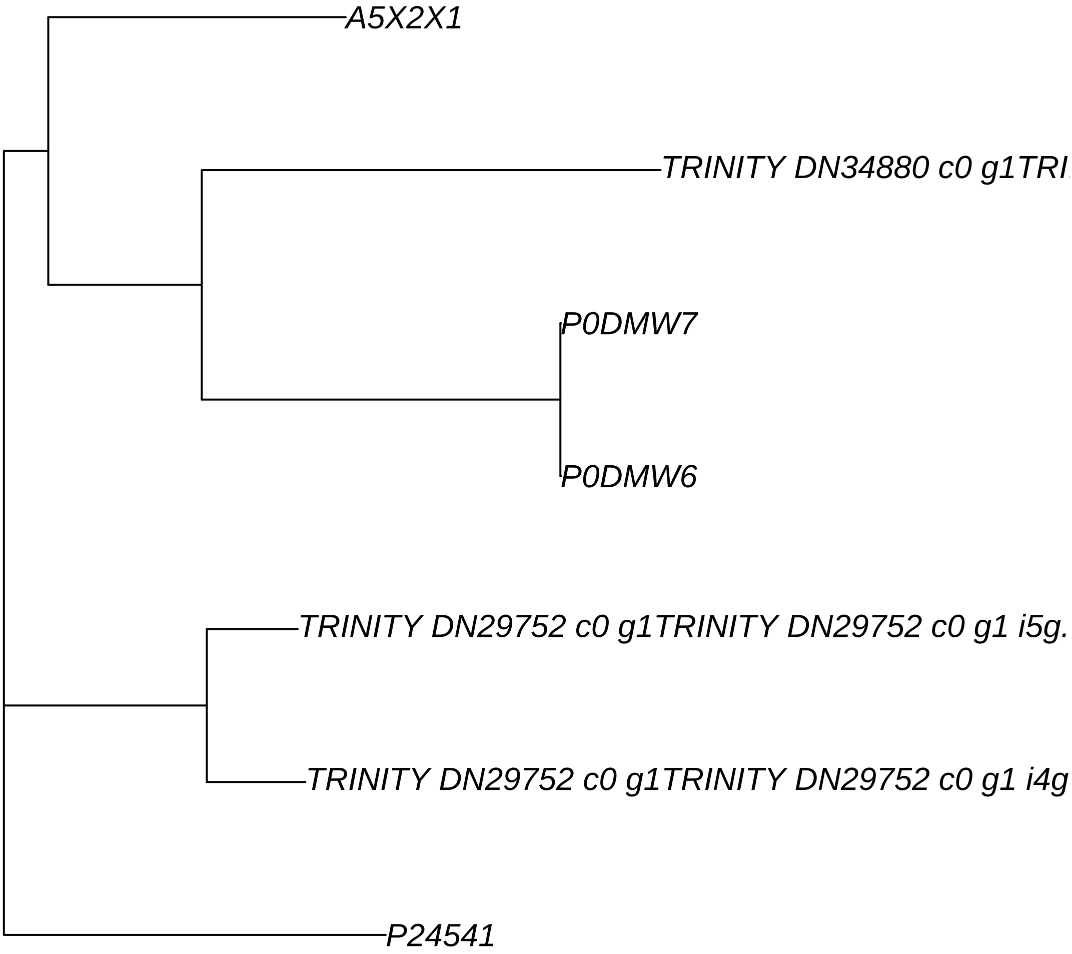
A5X2X1
TRINITY DN34880 c0 g1TRI
P0DMW7
P0DMW6
TRINITY DN29752 c0 g1TRINITY DN29752 c0 g1 i5g.
TRINITY DN29752 c0 g1TRINITY DN29752 c0 g1 i4g
P24541
